# Response to “Beyond Pain: Sarcopenia, Malnutrition, and Sex-Specific Body Image Distortion as Potential Additional Mediators of HRQOL Impairment in ADPKD”

**DOI:** 10.1016/j.ekir.2026.106680

**Published:** 2026-06-20

**Authors:** Jad Fadlallah, Matthew Ho, Muhammad T. Hassan, Taher Dehkharghanian, Mauricio Miranda Cam, Seung Heyck Lee, Saima Khowaja, Daniel G. Bichet, Pierre A. Brown, Matthew B. Lanktree, Louise Moist, Bill Wang, Korosh Khalili, Istvan Mucsi, York Pei

**Affiliations:** 1 Division of Nephrology, University Health Network and University of Toronto, Toronto, Ontario, Canada; 2 Division of Nephrology, University of Montreal, Montreal, Quebec, Canada; 3 Division of Nephrology, Department of Medicine, University of Ottawa, Ottawa, Ontario, Canada; 4 Division of Nephrology, McMaster University, Hamilton, Ontario, Canada; 5 Division of Nephrology, Western University, London, Ontario, Canada; 6 Patient Liaison Advisory Group of the Internation al Society of Nephrology, Hong Kong, China; 7 Department of Medical Imaging, University Health Network and University of Toronto, Toronto, Ontario, Canada

The Author Replies:

We thank Farinone *et al.*^1^ for their thoughtful letter commenting on our article on the association of kidney volume with patient-reported outcomes in autosomal dominant polycystic kidney disease.^2^ Their comments raise important questions regarding nutritional limitation, altered body composition, and sex-specific burden as contributors to impaired health-related quality of life (HRQOL).

Using available patient-reported data, we explored whether the ability to eat a full-sized meal without discomfort was associated with HRQOL. Patients able to do so had an adjusted physical component summary (PCS) score approximately 6.6 points higher than those unable to do so, exceeding the difference associated with kidney pain (Table 1). By contrast, this variable was not clearly associated with mental component summary (MCS), whereas kidney pain was strongly associated with both PCS and MCS. These findings suggest that abdominal or nutritional limitation may be particularly relevant to physical HRQOL. Notably, total kidney volume was associated with kidney pain in our original analysis,^2^ but not with full-meal discomfort in this exploratory analysis, suggesting that meal-related discomfort may reflect a broader abdominal, nutritional, or functional burden not captured by kidney volume alone.

**Table 1:** Exploratory analyses of nutritional limitation, adjusted BMI, and sex-disaggregated HRQOL in ADPKD

Panel A. Full-sized meal without discomfort, kidney pain, and HRQOL							
Exposure	Outcome	Analytic N	Adjusted mean: reference	Adjusted mean: comparison	Adjusted mean difference	95% CI	*P*-value
Full-sized meal without discomfort: Yes (*N* = 358) vs. No (*N* = 47)	PCS	405	46.12	52.77	+6.65	4.31–8.98	<0.001
	MCS	405	45.31	48.43	+3.12	−0.27 to 6.51	0.071
Kidney pain: Pain (*N* = 212) vs. No pain (*N* = 241)	PCS	456	53.97	49.98	−3.99	−5.43 to -2.55	<0.001
	MCS	456	50.33	45.63	−4.70	−6.72 to -2.68	<0.001


Panel B. KDIGO-adjusted BMI class and PCS, normal weight as reference							
Exposure	BMI class	Analytic N	Adjusted mean PCS	Reference mean PCS	Mean difference vs normal weight	95% CI	*P*-value
KDIGO-adjusted BMI	Underweight	10	49.01	54.09	−5.08	−10.11 to −0.05	0.048
	Normal weight	182	54.09	54.09	Reference	--	--
	Overweight	134	50.97	54.09	−3.12	−4.88 to −1.36	0.001
	Obesity	53	48.69	54.09	−5.40	−7.82 to −2.98	<0.001


Panel C. Sex differences in HRQOL after adjustment for continuous TKV							
Exposure	Outcome	Analytic N	Adjusted mean: Male	Adjusted mean: Female	Adjusted mean difference (Female - Male)	95% CI	*P*-value
Female (*N* = 240) vs. Male (*N* = 216)	PCS	456	52.85	51.39	−1.46	−2.95 to 0.03	0.054
	MCS	456	50.02	46.42	−3.60	−5.66 to −1.54	0.001

ADPKD, autosomal dominant polycystic kidney disease; BMI, body mass index; CI, confidence interval; HRQOL, health-related quality of life; KDIGO, Kidney Disease: Improving Global Outcomes; MCS, mental component summary; PCS, physical component summary; TKV, total kidney volume.

In panel A, the reference and comparison groups correspond to the comparison stated in the first column. Adjusted means and mean differences are from the corresponding regression/margins models. In exploratory analysis, TKV was not associated with the ability to eat a full-sized meal without discomfort (odds ratio: 0.99; 95% CI: 0.90 to 1.08; *P* = 0.70). In panel B, analytic *N* = 379. Mean differences compare each KDIGO-adjusted BMI class with normal weight. Regarding panel C, the TKV tertile × sex interaction for MCS was not statistically significant (*F*_2,427_ = 0.39; *P* = 0.679). In all panels, models adjusted for sex, estimated glomerular filtration rate, age, ethnicity, and continuous TKV.

We also examined a Kidney Disease: Improving Global Outcomes 2025–aligned adjusted body mass index measure that accounts for kidney- and liver volume–derived weight.^3^ Normal adjusted body mass index was associated with the highest PCS, whereas both underweight and obesity were associated with lower PCS (Table 1). These exploratory findings are compatible with a nutritional or body composition pathway linking organomegaly to physical HRQOL but require confirmation using direct measures of nutritional status, muscle mass, and body composition.

Finally, we performed sex-disaggregated analyses. Female participants had similar PCS but significantly lower MCS than male participants, and this difference persisted after adjustment for total kidney volume, estimated glomerular filtration rate, age, and ethnicity (Table 1). The total kidney volume tertile × sex interaction for MCS was not statistically significant, indicating no clear evidence that the total kidney volume–MCS association differed by sex. Overall, our findings suggest a sex-related mental HRQOL burden among women with autosomal dominant polycystic kidney disease that warrants further study.

## Disclosure

All the authors declared no conflict of interests.
